# A Loop-Mediated Isothermal Amplification Assay and Sample Preparation Procedure for Sensitive Detection of *Xanthomonas fragariae* in Strawberry

**DOI:** 10.1371/journal.pone.0147122

**Published:** 2016-01-14

**Authors:** Hehe Wang, William W. Turechek

**Affiliations:** U.S. Department of Agriculture-Agricultural Research Service (USDA-ARS), U.S. Horticultural Research Laboratory, Fort Pierce, FL, United States of America; National Cheng Kung University, TAIWAN

## Abstract

*Xanthomonas fragariae* is a bacterium that causes angular leaf spot of strawberry. Asymptomatic infection is common and contributes to the difficulties in disease management. The aim of this study was to develop a loop-mediated isothermal amplification (LAMP) assay as an efficient method for detection of asymptomatic infections of *X*. *fragariae*. In addition, a new method of sample preparation was developed that allows sampling of a larger amount of plant tissue, hence increasing the detection rate in real-life samples. The sample preparation procedure includes an overnight incubation of strawberry tissues in phosphate-buffered saline (PBS), followed by a quick sample concentration and a boiling step to extract DNA for amplification. The detection limit of the LAMP assay was approximately 2×10^3^ CFU/mL for pure bacteria culture and 300 CFU/mL for bacteria spiked strawberry leaf and petiole samples. LAMP provided a 2–3 fold lower detection limit than the standard qPCR assay but was faster, and more user-friendly. The LAMP assay should serve as a rapid, sensitive and cost-effective tool for detecting asymptomatic infections of *X*. *fragariae* in strawberry nursery stock and contribute to improved disease management.

## Introduction

*Xanthomonas fragariae* is a bacterium that causes angular leaf spot (ALS) of strawberry. It is an important disease in strawberry production regions worldwide, particularly in nursery production [1–3]. The bacterium attacks mainly the foliage and calyx of the plant and symptoms on these tissues are typically diagnostic. Infected plants first develop water-soaked, semi-translucent angular lesions on the lower leaf surface that eventually appear as reddish-brown irregular spots on the upper leaf surface, usually coalescing to form larger patches that may or may not be accompanied by a diffuse yellowing of the surrounding leaf tissue. The pathogen is transmitted from nursery- to fruit-production fields mainly through latent infections on asymptomatic stock. When the pathogen becomes active, secondary spread in the field occurs largely through splashing water. Plantings are particularly vulnerable to ALS in regions where rain is prevalent during the production season or where overhead irrigation is used for plant establishment and/or frost protection. Currently, there are no commercial strawberry varieties that are immune to disease and no effective chemical treatments are commercially available.

ALS significantly affects the commercial value of the crop in several ways. In nursery production, losses occur when plant batches are rejected by inspectors due to violations of quarantine regulations. For example, to export strawberry plants to Europe, the European and Mediterranean Plant Protection Organization (EPPO) mandates that nurseries maintain phytosanitary standards that includes planting material to be derived from mother plants certified free of *X*. *fragariae*, and production sites be documented free from ALS for the past five growing seasons [1, 3]. In domestic fruit production, ALS can reduce fruit yield when infection is severe, and unsightly infections of the calyx can render fruit unmarketable. Based on estimates from the nursery industry, annual production losses due to ALS range from 5–8% industry-wide, valued between ~$700,000 and $1,100,000. For individual nurseries, losses can be as low as 2% to as high as 30%.

ALS has become increasingly problematic over the past ten years, likely due to a combination of the bacteria becoming entrenched in some production settings, our inability to accurately diagnose infected nursery stock, and the absence of effective disease management strategies. One of the factors that makes management particularly difficult is that *X*. *fragariae* can survive at low densities on plants without producing symptoms, thus complicating our ability to identify infected plants and remove them from the production chain. To address this issue, it is necessary to develop an user-friendly and reliable assay capable of detecting low quantities of *X*. *fragariae* contamination in nursery stock before they go into planting. To this end, several molecular detection tools have been developed for *X*. *fragariae* over the past twenty years. These include an enzyme linked immunosorbent assay (ELISA) [4], conventional PCR [5], nested-PCR [6–8], and Taqman-PCR [9–11]. ELISA was found to cross-react with some other *Xanthomonas* species [12], and among the PCR assays, Taqman-PCR has been shown to be the fastest and most sensitive.

In recent years, loop-mediated isothermal amplification (LAMP) has emerged as a popular tool for pathogen detection in different phytopathological systems, including several with Xanthomonas species [13–22]. In contrast to PCR, LAMP amplifies at a single, constant temperature and therefore does not require a sophisticated and expensive thermal cycler to conduct the assay, which makes the test more convenient and less expensive to implement [23]. In general, LAMP assays consist of a set of six primers that recognize eight regions on the target DNA to yield high specificity. In addition, LAMP reactions can amplify much faster than PCR and are thought to be less sensitive to inhibitors that affect PCR [24]. Positive LAMP samples can be visualized by gel electrophoresis or by adding DNA-intercalating dyes after the reaction; however, these methods require exposure of the amplicons post-reaction which may lead to cross-contamination among samples due to the high level of amplification in LAMP and cause false-positive results. Instead, adding hydroxynaphthol blue (HNB) dye to the reaction mix prior to running the test has been found to eliminate cross contamination while efficiently distinguishing between positive and negative samples [25]. LAMP can also be monitored in real-time by measuring the increase in turbidity [20, 26], by adding fluorescent dyes prior to the reactions [22], or even by immunoassay-based detection with labeled primers [19, 21]. With the advantage of viewing results in a multitude of formats, LAMP is feasible for both field- and lab-based pathogen detection.

One limitation of many DNA-based detection methods is that plant samples are required to be purified through use of commercial DNA extraction kits which usually allow only small amounts of tissue to be sampled, or the more tedious, technically challenging, and hazardous phenol-chloroform method. Development of a method that allows a greater proportion of tissue to be sampled and increase the likelihood of detection could greatly improve our ability to detect ALS in nursery stock.

The aim of this study was to develop a more cost-effective and user-friendly method to detect *X*. *fragariae* in strawberry plants. A LAMP assay was developed and was coupled with a new sample preparation procedure that included a sample incubation step and a simple boiling protocol for DNA extraction. The incubation procedure was developed to facilitate detection when low densities of the bacteria are expected. Latent class analysis (LCA) was used to evaluate the performance of the real-time LAMP assay with fluorescent dyes. The performance of the LAMP assay with HNB dye for end product detection was also characterized. The latter method may serve as a more convenient method for on-site diagnosis.

## Materials and Methods

### Preparation of *X*. *fragariae* cultures

*X*. *fragariae* isolates were grown on sucrose-peptone agar (SPA) for 3 to 7 days at room temperature, then transferred to sucrose-peptone broth (SPB) on an orbital table shaker to grow for an additional 24–48 h [27]. The bacterial suspensions were centrifuged at 3,000 *g* for 3 min, re-suspended in 1x phosphate buffered saline (PBS), and adjusted to an optical density (OD_620_) of 0.1, which is equivalent to ~10^8^ CFU/mL [28]. Samples were further diluted with 1x PBS into the following concentrations: 2×10^3^, 1×10^3^, 6×10^2^, 4×10^2^, and 1×10^2^ CFU/mL. Aliquots of each dilution (100 μL) were plated on SPA plates and were counted 5 days after plating to confirm bacterial densities.

### Sample preparation from strawberry tissue

A simple sample preparation procedure was developed to increase the likelihood of detection of *X*. *fragariae* in leaf and petiole tissue for situations where the bacterial population is expected to be low and unevenly distributed. Four candidate media were chosen for testing using past experience and a cursory review of the literature as a guide for their selection [1, 10, 27, 29]. The selected media were PBS, distilled water (H_2_O), SPB, and R2A broth (Teknova, Hollister, CA). We use the term media broadly to refer to substances that may or may not contain nutrients. Several experiments were conducted to identify which of these media would yield the best detection results.

#### (i) Media selection based on bacteria-spiked tissue samples

About 1 g of healthy leaf or petiole tissue from strawberry variety Festival were cut with a clean razor blade in ~5 and 2 mm^2^ pieces, respectively, and added separately with a 100 μL aliquot of a suspension of *X*. *fragariae* strain Xf100 in PBS (10^6^ CFU/mL) to 10 mL of each candidate media in 50-mL sterilized flasks. Flasks were covered with aluminum foil and placed on an orbital shaker overnight. At 21 h after incubation, 1 mL of the media was collected per sample and processed for DNA extraction and tested by both LAMP and quantitative (q)PCR (both described below) to identify the media best suited for detection. Media containing leaf and petiole tissue without added bacteria served as negative controls. Each sample had three replicates. The experiment was repeated three times.

#### (ii) Media selection based on inoculated tissue samples

Strawberry plants from six varieties (Benicia, Chandler, Festival, Monterey, Portola, and Radiance) were inoculated with a 10^6^ CFU/mL water suspension of *X*. *fragariae* strain Xf100. Seven days after inoculation (dai), 1 g samples of tissue from asymptomatic leaves (all leaves were asymptomatic) were collected, processed as above, and incubated in 10 mL of each of the four media for 21 h. A 1 mL sample was drawn from each flask after the incubation period and processed for DNA extraction and tested by both LAMP and qPCR (both described below) to identify the media best suited for detection. There were two replicate samples per variety/media and replicates were run over time. Pure media served as the negative controls.

#### (iii) Measuring the effect of incubation in the sample preparation procedure on downstream DNA amplification

**Fig 1:**
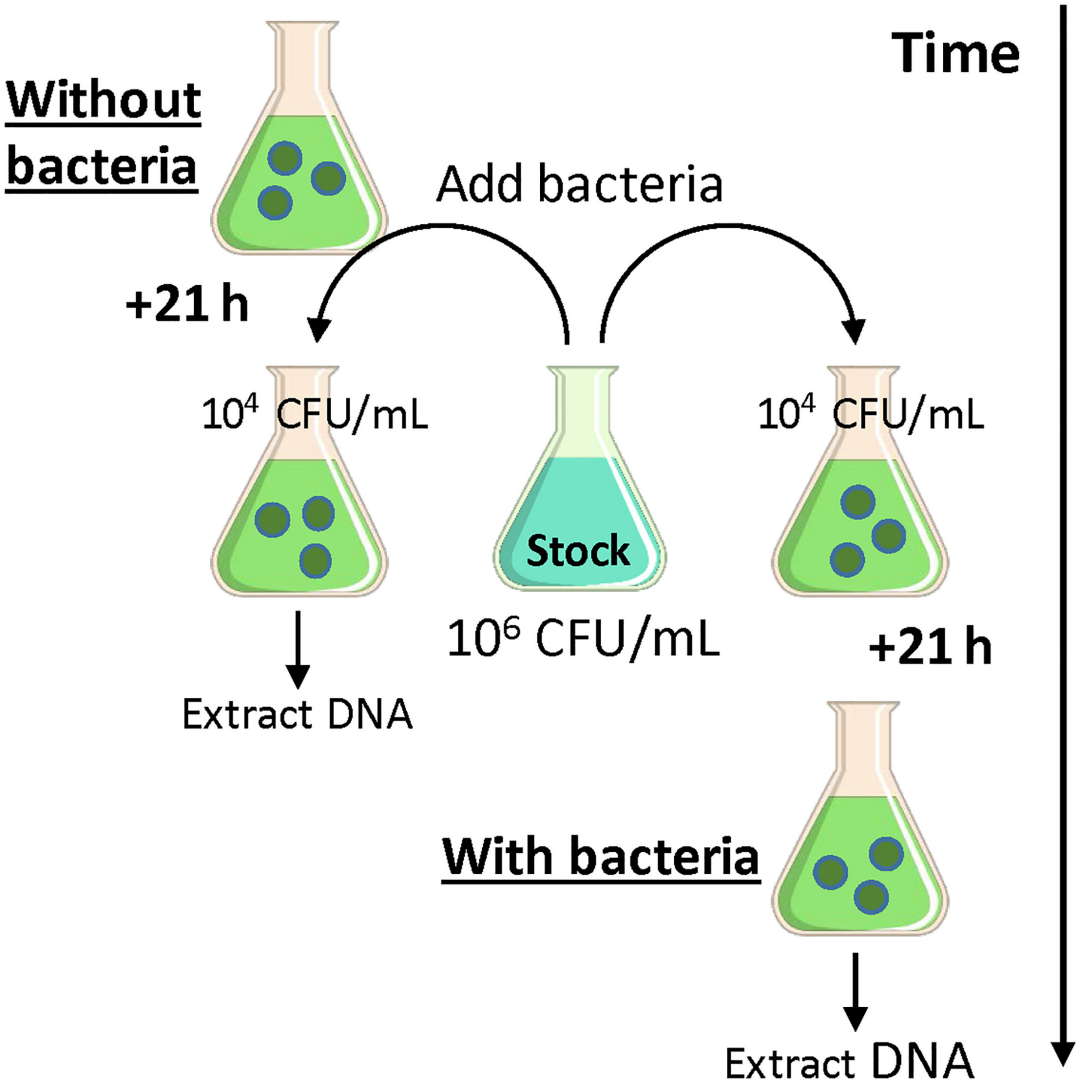
Experimental outline for measuring how incubating strawberry tissue (dark circles within each flask) in the sample preparation procedure affects downstream DNA amplification.

This experiment was designed to determine whether the 21 h incubation step of the sample preparation procedure enhanced detection of *X*. *fragariae* in downstream LAMP and qPCR amplification. First, to examine the effect of incubating tissue alone (without added bacteria) on DNA amplification, 1 g of healthy ‘Festival’ leaf or petiole tissue (processed as above) was incubated in 10 mL of each media in 50-mL sterilized flasks for 21 h at room temperature (Fig 1). After the 21 h incubation period, 1 mL of each media was transferred to 2 mL centrifuge tubes and an aliquot of Xf100 suspension–from a stock culture of 10^6^ CFU/mL Xf100 suspension in PBS–was added to create a final concentration of 10^4^ CFU/mL. Samples were immediately processed for DNA extraction and tested by both LAMP and qPCR as described below. Coinciding with the end of the first 21 h incubation period, a second set of flasks containing 10 mL of PBS and 1 g of healthy ‘Festival’ leaf or petiole tissue, were spiked with a 100 μL aliquot of an Xf100 suspension from the same stock culture to create a starting bacterial concentration of 10^4^ CFU/mL and allowed to incubate for 21 h at room temperature (Fig 1). After the incubation period, 1 mL of media was collected per sample, processed for DNA extraction, and tested by both LAMP and qPCR as described below. Negative controls for each set of samples were prepared identically except without added bacteria. There were three replicate flasks for each treatment. The experiment was conducted twice.

#### (iv) Evaluation of the sample preparation procedure across X. fragariae strains and strawberry varieties

PBS was selected as the optimal medium based on results obtained from the experiments outlined above (see Results). The final procedure was tested on combinations of four strawberry varieties (Camarosa, Festival, Portola, and Ventana) and seven strains of *X*. *fragariae* (Xf100, Xf101, Xf128, Balm, Xf1425, Xf1429, and Xf1431) to determine the robustness of the procedure. For each combination of strawberry variety and strain of *X*. *fragariae*, 100 μL of a 10^6^ CFU/mL bacterial-PBS suspension was added into the incubation flasks containing 10 mL of PBS and 1 g of fresh leaf or petiole tissue processed as described above. Leaf or petiole tissue incubated in PBS buffer alone served as negative controls. Samples were collected at both 3 h and 21 h after incubation to determine if the 21 h incubation period could be shortened. The experiment was conducted twice with leaf tissue and once with petiole tissue. There were two replicated samples for each variety/strain combination in each of the three experiments. DNA was extracted and tested by both LAMP and qPCR as described below.

#### (v) Comparing detection rates between samples processed by the new sample preparation procedure and the standard technique

Twenty-seven ‘Festival’ plants were spray-inoculated with either a 10^4^, 10^3^, or 10^2^ CFU/mL water suspension of *X*. *fragariae* strain Xf100; nine plants per inoculum concentration. At 20, 30, and 50 dai, 18, 27, and 33 leaf and petiole samples were collected from these plants, respectively. Each sample was processed with both the QIAGEN DNeasy Plant Mini Kit (QIAGEN, Valencia, CA)–generally our standard method for processing samples–and through the sample preparation procedure developed in this study.

For standard processing (i.e., DNA extraction with the QIAGEN kit), 100 mg of tissue was macerated with two nickel plated steel balls (K&J magnetics Inc., Pipersville, PA) in 600 μL of AP1 buffer mixed with 4% of PVP-40 (Sigma-Aldrich, St. Louis, MO) using a Mini Beadbeater-96 (Biospec products Inc., Bartlesville, OK). After incubation at 65°C for 10 min, samples were mixed with 180 μL of P3 buffer, processed following manufacturer’s protocol, and then eluted in 100 μL of AE buffer. For samples processed through the sample preparation procedure, DNA was extracted via the Triton-boiling approach (described below) and was suspended in 100 μL of 1% Triton X-100. A 1 μL aliquot of DNA from each method was tested by LAMP and qPCR (both described below) to compare which of these methods was best suited for detection of *X*. *fragariae*.

#### (vi) DNA extraction

The method of DNA extraction for the sample preparation procedure was a modified version of the Triton-boiling approach developed by Sowmya et al. [30]. In the modified protocol, 1-mL samples of each media were collected and centrifuged at 20,000 *g* for 5 min. After discarding the supernatant, the pellet was re-suspended in 100 μL of 1% Triton X-100 by vortex. Cell suspensions were then boiled for 10 min, cooled at room temperature for 5 min, and frozen at -20°C for 10 min. Frozen samples were thawed at room temperature for 5 min and centrifuged at 12,000 *g* for 3 min. The supernatant contained the DNA template used in downstream LAMP and qPCR reactions.

### LAMP primer design and reaction conditions

LAMP primers were designed to target the genomic sequence of RAPD marker 295 of *X*. *fragariae* (GenBank accession No. EU176816.1) [5, 10]. The marker 295 was chosen because it was one of the previously established markers for *X*. *fragariae* detection and it was highly conserved among *X*. *fragariae* strains tested in previous studies and this current study [5, 10]. The Taqman PCR primers for marker 295 (used in this study for comparison with LAMP) were shown to be a highly sensitive and specific set of primers among other Taqman primers for *X*. *fragariae* detection [10]. Primer sequences were generated using PrimerExplorer V4 software (Eiken Chemical Co. Ltd., Tokyo, Japan, http://primerexplorer.jp/e/), and then searched in Genbank databases by BLAST for specificity (Table 1).

**Table 1:** LAMP primers for amplification of the RAPD marker 295 region of *Xanthomonas fragariae*.

Primer	Nucleotide Sequence (5' → 3')
Forward outer (F3)	GGGTTTTTTCCAAGGCCGTA
Backward outer (B3)	TCGATCGTAAGCGATGGTCT
Forward Inner (FIP)	TGGGCTCGCCAACTGTCAACTGCCGAATACGACTGGATGA
Backward Inner (BIP)	GCAGAGGCACCGGATCTTAGTTGAGGCGAGCTCATTAAGACG
Forward Loop (LF)	ATTGCATCAGTCCGCTTTGGG
Reverse Loop (LB)	TGAAAGCGAAATGCGAAATTTTCGG

LAMP reactions were optimized through testing of different fluorescent dyes (SYBR^®^ Green I and SYTO^®^9, Invitrogen, Grand Island, NY), adjusting the concentration of dNTPs, primers, MgSO_4_, and PCR additives (DMSO and Betaine). The final 20 μL LAMP reaction consisted of 0.2 μM each of outer primers (F3 and B3), 1.6 μM each of inner primers (FIP and BIP), and 0.8 μM each of loop primers (LF and LB), 2 μM of SYTO^®^9 or 125 μM of hydroxynaphthol blue (HNB, Sigma-Aldrich, St. Louis, MO), 1.2 mM of dNTPs, 6 mM of MgSO_4_, 1x isothermal amplification buffer, 8 U of Bst2.0 WarmStart DNA polymerase (New England Bio Labs, MA, USA), and 1 μL of template DNA. The real-time LAMP reactions with SYTO^®^9 were conducted in an Eppendorf Realplex^4^ master cycler (Eppendorf North America, Hauppauge, NY) at 65°C for 45 min. Melting curve analysis was conducted at 95°C for 15 s, followed by an increment from 78 to 98°C at 0.04°C /s. To view the LAMP products in agarose gel, 4 μL of the real-time LAMP amplicons were run in 1.5% of agarose gel with a 100 bp DNA ladder. The gel was stained with ethidium bromide (EtBr) for visual detection with a UV illuminator. The LAMP reactions with HNB dye were observed for color change from violet to sky blue for positive samples at 35 min after incubation at 65°C. Non-template controls were included in each run. Each sample was run in three technical replicates.

### qPCR reaction conditions

Taqman PCR primers q295 developed previously for *X*. *fragariae* detection [10] were used exclusively in this study. The 20 μL qPCR reaction contained 0.36 μM of forward and reverse primers, 0.17 μM of the Taqman probe, and 1x master mix from the DyNAmo Flash Probe qPCR Kit (Thermo Fisher Scientific, Pittsburgh, PA). The reactions were run in an Eppendorf Realplex^4^ master cycler at 95°C for 7 min, followed by 40 cycles of 95°C for 10 s and 55°C for 30 s. Non-template control was included in each run. Each sample was run in two technical replicates.

### Amplification specificity of LAMP and qPCR primers

Both LAMP and qPCR primers were tested with 40 *X*. *fragariae* isolates, 17 isolates of other *Xanthomonas* species, 29 isolates of different strawberry pathogens, 15 unclassified bacterial and fungal isolates from healthy strawberry tissues, and 5 *Pseudomonas* isolates from other hosts (Table 2). DNA from bacterial isolates (~10^8^ CFU/mL for *X*. *fragariae* isolates and ~10^9^ CFU/mL for the others) was extracted using the modified Triton boiling method described above, while fungal DNA was extracted using QIAGEN’s DNeasy Plant Mini Kit (QIAGEN, Valencia, CA) following manufacturer’s protocol.

**Table 2:** Bacterial and fungal isolates used for testing amplification specificity and their corresponding qPCR and LAMP results^a^.

Species/Isolates	# of isolates	Host	qPCR	LAMP
*Alternaria* spp.	1	Strawberry	40.0	45.0
*Botrytis cinerea*	3	Strawberry	40.0	45.0
*Cercospora* spp.	1	Strawberry	40.0	45.0
*Colletotrichum acutatum*	1	Strawberry	40.0	45.0
*Fusarium oxysporum*	1	Strawberry	40.0	45.0
*Gnomonia* spp.	2	Strawberry	39.0	45.0
*Hainesia lythri*	1	Strawberry	38.5	45.0
*Macrophomina phaseoli*	2	Strawberry	39.7	45.0
*Pestalotiopsis* spp.	2	Strawberry	40.0	45.0
*Phomopsis obscurans*	3	Strawberry	40.0	45.0
*Phytophthora cactorum*	3	Strawberry	40.0	45.0
*Pseudomonas cichorii*	1	Tomato	40.0	41.1
*Pseudomonas putida*	1	Tomato	37.7	41.5
*Pseudomonas syringae pv*. *syringae*	1	Bean	37.5	45.0
*Pseudomonas syringae pv*. *tomato*	1	Tomato	40.0	45.0
*Pseudomonas viridiflava*	1	Tomato	37.8	39.9
*Psuedomonas* spp.	4	Strawberry	37.7	45.0
*Psuedomonas syringae*	1	Strawberry	38.0	40.0
*Pythium irregulare*	1	Strawberry	40.0	45.0
*Verticillium dahliae*	3	Strawberry	39.1	45.0
*Xanthomonas albilineans*	1	Sugar cane	38.2	45.0
*Xanthomonas alfalfae*	1	Citrus	40.0	40.5
*Xanthomonas arboricola-pruni*	1	Peach	38.7	39.7
*Xanthomonas axonopodis pv*. *dieffenbachiae*	1	Anthurium	39.1	40.8
*Xanthomonas campestris pv*. *citri*	1	Citrus	40.0	45.0
*Xanthomonas campestris pv*. *campestris*	1	Cabbage	38.1	41.5
*Xanthomonas campestris pv*. *euvesicatoria R4*	1	Pepper	39.9	45.0
*Xanthomonas codiaei*	1	Croton	38.3	40.0
*Xanthomonas fragariae*	40	Strawberry	21.8	7.9
*Xanthomonas fuscans*	1	Bean	38.8	39.3
*Xanthomonas hortorum pv*. *pelargonii*	1	Geranium	37.3	40.6
*Xanthomonas hyacinthi*	1	Hyacinthus	37.7	44.7
*Xanthomonas perforans T4*	1	Tomato	40.0	45.0
*Xanthomonas pisi*	1	Pea	37.4	39.1
*Xanthomonas* spp.	1	Unknown	39.2	40.3
*Xanthomonas translucens*	1	Wheat	37.2	41.2
*Xanthomonas vesicatoria*	1	Tomato	39.4	40.3
*Xanthomonas vesicatoria*	1	Pepper	39.0	39.8
Unclassified	15	Strawberry	40.0	45.0

^a^ qPCR and LAMP data are presented as the means of the C_t_ and T_t_ values per microbial species/isolates for qPCR and LAMP, respectively.

### Detection limits of LAMP and qPCR primers

#### (i) Pure bacterial culture

To determine the minimum detectable concentration of *X*. *fragariae* in pure culture, a serial dilution of *X*. *fragariae* strain Xf100 suspension in 1% triton was prepared to achieve 10^8^, 10^7^, 10^6^, 10^5^, 10^4^, 6×10^3^, 3×10^3^, 2×10^3^, and 1×10^3^ CFU/mL. DNA from 100 μL of each cell suspension was extracted by adding 1 μL of 100% Triton X-100 and following the modified Triton-boiling method described above, starting with the boiling step. The experiment was repeated three times, with three biological replicates per experiment.

#### (ii) Bacteria-spiked strawberry samples

To determine the detection limit from samples processed through the sample preparation procedure, 100 μL of *X*. *fragariae* strain Xf100 suspension in PBS was added into 10 mL PBS at concentrations of 1×10^6^, 1×10^5^, 6×10^4^, 3×10^4^, 2×10^4^, 1×10^4^, and 6×10^3^ CFU/mL with 1 g of healthy leaf or petiole tissue from ‘Festival’. Healthy leaf or petiole tissues in PBS alone were included in each experiment as negative controls. Samples (1 mL each) were collected at 21 h after incubation. There were three replicates for each sample and the experiment was conducted three times. DNA samples were tested by both LAMP and qPCR.

### Sensitivity and specificity of the LAMP assay from field samples

Strawberry leaf and petiole samples were collected from: a) commercial strawberry fields located in Balm, FL with widespread, visible symptoms of ALS; b) experimental field plots at the University of Florida’s Gulf Coast Research (GCREC) and Education Center in Wimauma, FL with very low incidence of ALS; and c) field plots without detectable ALS located at the US Horticultural Research Laboratory (USHRL) in Fort Pierce, FL. Samples from commercial fields were collected from the variety Radiance; however, fields differed in that plants were purchased from different nurseries in North America, i.e., Canadian, California, and North Carolina. Samples from the experimental field plots were collected from ‘Festival’ and ‘Ventana’. Samples were collected to have approximate even representation of leaves and petioles from symptomatic and asymptomatic plots. Samples from symptomatic plots contained leaves/petioles with and without visible ALS symptoms. A total of 147 matching leaf and petiole samples were processed through the sample preparation procedure and tested for *X*. *fragariae* by both LAMP and qPCR as described above. A total of 293 test results were obtained (one petiole sample was lost). Of these, 123 samples were from the commercial operation, and 150 and 20 samples were collected from the experimental plots at USHRL and GCREC, respectively. Results were used for determining the sensitivity and specificity of the LAMP and qPCR assays.

### Statistical analyses

#### (i) Media selection for the sample preparation procedure

For the experiment based on bacteria-spiked tissue samples, the response variables for qPCR (C_t_, threshold cycle) and LAMP (T_t_, threshold time) were analyzed in separate general linear mixed models (GLMM) using the SAS procedure GLIMMIX. Media and tissue type were treated as crossed fixed effects, and replication, experiment, and experiment’s interactions with the fixed effects were treated as random effects. An identity link function and Gaussian (normal) error distribution were specified. Treatment differences were obtained using the LSMEANS statement with the LINES option. For the experiment based on inoculated plant tissues, the response variables C_t_ and T_t_ were analyzed in a general linear model (GLM) treating media and variety as crossed fixed effects, specifying an identity link function and Gaussian (normal) error distribution, using the SAS procedure GLIMMIX. Treatment differences were obtained using the LSMEANS statement with the LINES option.

#### (ii) Measuring the effect of the incubation in the sample preparation procedure on downstream DNA amplification

DNA amplification from tissue samples incubated for 21 h with and without added bacteria (Fig 1) were characterized by both C_t_ and T_t_ and results were analyzed in separate GLMMs using the SAS procedure GLIMMIX. As above, media and tissue type were treated as crossed fixed effects, and replication, experiment, and experiment’s interactions with the fixed effects as random effects. An identity link function and Gaussian (normal) error distribution were specified. To evaluate the effect of the incubation step on detection in each of the media, the differences in C_t_ or T_t_ values between samples incubated with and without bacteria were obtained and analyzed by paired *t*-tests.

#### (iii) Evaluation of the sample preparation procedure across X. fragariae strains and strawberry varieties

The response variables C_t_ and T_t_ were analyzed in GLMMs using the SAS procedure GLIMMIX, treating variety and *Xanthomonas* strain as crossed fixed effects, and replication, experiment, and experiment’s interactions with the fixed effects as random effects. An identity link function and Gaussian (normal) error distribution were specified. Treatment differences were obtained using the LSMEANS statement with the LINES option. The 3 h and 21 h results were analyzed separately for both C_t_ and T_t_. Paired *t*-tests were used to evaluate the difference in C_t_ or T_t_ values between the 3 h and 21 h results combined across all factor levels.

#### (iv) Comparing detection rates between samples processed by the new sample preparation procedure and the standard technique

The response variables C_t_ and T_t_ were analyzed in a GLM treating the independent variables procedure, tissue, sampling time (dai) and inoculum concentration as fixed, categorical effects. An identity link function and Gaussian (normal) error distribution were specified. Treatment differences were obtained using the LSMEANS statement with the LINES option. The analysis was done using the SAS procedure GLIMMIX.

#### (v) Sensitivity and specificity of the LAMP assay from field samples

Latent class analysis (LCA) was used to estimate the sensitivities and specificities of the LAMP and qPCR assays based on results obtained from testing of the field samples, although it is the characteristics of the LAMP assay that is of immediate interest. The sensitivity of a test is defined as the conditional probability of a positive test result given the sample is truly positive, while the specificity is the conditional probability of a negative test result given the sample is truly negative. The latent class approach is distinguished from other methodologies used to evaluate diagnostic tests in that it does not require results from a gold standard test (i.e., a perfect test) as a reference. Instead, LCA exploits the cross-classified results from two or more tests and uses a maximum likelihood approach to designate individual test results into two mutually exclusive categories (+ or −) and then uses this information to estimate each test’s characteristics. A detailed description of the procedure in a plant pathology setting can be found in Turechek et al. [31].

The LCA model was constructed to assume that both tests produced a binary outcome; pathogen presence or absence. Therefore, cutoff or threshold values were constructed to produce a binary result even though both C_t_ and T_t_ are continuous variables. For example, if C_t_ = 35 was selected as a cutoff, then samples with C_t_ > 35 would be classified as pathogen free and samples with C_t_ ≤ 35 would be classified as positive for *X*. *fragariae*. However, because threshold selection is subjective and dependent upon the application, test performances were characterized initially for all combinations of qPCR C_t_ values between 30 and 40 cycles and LAMP T_t_ values between 22 and 32 min, in increments of 1 and 2 min, respectively. A narrower range of threshold values in smaller increments (0.1 and 0.2) was investigated between cutoff values where qPCR sensitivity and specificity were equal to one. A categorical variable was necessarily created to increase the models degrees of freedom to allow estimation of two sensitivities, two specificities, and the incidence of disease in the two groups created by the categorical variable [31]. Sample location was designated the categorical variable with samples collected from the commercial operation serving as one level and samples collected from research farms serving as the second level of the variable. Estimated sensitivities and specificities were used to calculate Youden’s index, a generic index used to identify optimal threshold values. The threshold identified by this index represents a balance between the sensitivity and specificity of the test and is calculated by the formula: sensitivity + specificity– 1.

The latent class analysis was conducted using the SAS procedure LCA [32, 33] and a SAS macro was written by the authors to perform the analyses and extract the relevant information for the numerous threshold combinations. PROC LCA is an add-on procedure available through the Pennsylvania State University Methodology Center for SAS version 9.3 (SAS Institute, Cary, NC). All other calculations were done using Minitab v. 16 (Minitab Inc., State College, PA) and Sigma Plot v. 11 (San Jose, CA).

## Results

### Sample preparation procedure

#### (i) Media selection

For the bacteria-spiked samples, the effect of media on detection of *X*. *fragariae* at 21 h after incubation was significant according to qPCR and LAMP (*p* ≤ 0.0007); tissue type (i.e., leaf or petiole) did not have an effect on detection (Table 3). Among the four media tested, consistently lower C_t_ or T_t_ values were found with *X*. *fragariae* samples incubated in PBS. Similarly, for spray-inoculated leaves, the effect of media on detection of *X*. *fragariae* at 21 h after incubation was also significant according to both qPCR and LAMP (*p* < 0.0001, Table 3), with lower C_t_ or T_t_ values being found with leaves incubated in PBS. The variety of strawberry did not have an effect on detection according to qPCR and LAMP.

**Table 3:** Effect of media in the sample preparation procedure on *X*. *fragariae* detection after 21 h incubation^a^.

	Bacteria-spiked				Inoculated tissue			
	qPCR		LAMP		qPCR		LAMP	
Media	LS-means		LS-means		LS-means		LS-means	
H_2_O	36.8	A	40.5	A	39.5	A	45.0	A
SPB	36.8	A	36.0	A	34.5	B	42.3	B
R2A	32.7	B	20.4	B	35.0	B	45.0	A
PBS	30.5	C	12.5	C	33.7	C	21.9	C

^a^ Shown are the LS-means of the C_t_ (qPCR) and T_t_ (LAMP) values for each media at 21 h after incubation of 10^4^ CFU/mL of *X*. *fragariae* with healthy strawberry leaf or petiole tissues (bacteria-spiked) and 21 h after incubation of spray-inoculated strawberry leaf samples.

The standard errors are 1.02 (qPCR) and 2.81 (LAMP) for the bacteria-spiked samples, and 0.225 (qPCR) and 0.725 (LAMP) for the inoculated samples.

#### (ii) Measuring the effect of incubation in the sample preparation procedure on downstream DNA amplification

The effect of incubating strawberry tissue alone (Fig 1, “without added bacteria”) on DNA amplification was significant according to both qPCR (*p* = 0.0118) and LAMP (*p* < 0.0001) (Table 4), with samples in SPB and H_2_O demonstrating relatively greater inhibitory effects on amplification compared to samples in R2A and PBS. The effect of media on *X*. *fragariae* detection in bacteria-spiked strawberry samples at 21 h after incubation (Fig 1, “with added bacteria”) was also significant (as expected) according to qPCR (*p* = 0.0012) and LAMP (*p* = 0.0052). Tissue type did not have a significant effect on DNA amplification in either treatment. Based on the differences of C_t_ or T_t_ values between samples incubated with and without added bacteria, the 21 h incubation of bacteria-spiked strawberry tissues in PBS and SPB enhanced detection by C_t_’s of 1.2 and 1.4 (more than 2 fold differences in bacterial concentration based on the qPCR standard curve), respectively, and T_t_’s of 1.2 and 6.2, respectively (Table 4). However, only PBS showed statistically significant enhancement based on paired *t*-tests (qPCR: *p* < 0.001, LAMP: *p* = 0.002). Despite highest average differences in C_t_ or T_t_ values, the statistical outcome for samples incubated in SPB was affected by the greater variability among replicates.

**Table 4:** Quantification of the effect of incubating strawberry tissues with or without *X*. *fragariae* in the sample preparation procedure on downstream DNA amplification.

	Incubation without *X*. *fragariae*^a^				Incubation with *X*. *fragariae*^b^				Effect of incubation on detection^c^					
	qPCR		LAMP		qPCR		LAMP		qPCR t-test			LAMP t-test		
Media	LS-means		LS-means		LS-means		LS-means		Mean	SE	*P* value	Mean	SE	*P* value
H_2_O	35.2	A	33.4	B	35.9	A	38.8	A	-0.7	0.650	0.260	-5.4	2.950	0.097
SPB	37.2	A	40.0	A	35.7	A	33.8	A	1.4	0.661	0.051	6.2	3.154	0.076
R2A	32.0	B	14.2	C	31.3	B	16.4	B	0.7	0.426	0.112	-2.2	2.047	0.308
PBS	31.6	B	13.1	C	30.4	B	11.9	B	1.2	0.448	<0.001	1.2	0.295	0.002

^a^ Shown are the effect of incubated strawberry tissue suspension (without added bacteria) in each media on DNA amplification. The data presented are the LS-means of the C_t_ (qPCR) and T_t_ (LAMP) values of samples prepared from *X*. *fragariae* (10^4^ CFU/mL) freshly mixed with each media suspension (incubated with healthy strawberry tissues alone for 21 h) (Fig 1). The standard errors are 0.731 and 1.77 for qPCR and LAMP, respectively.

^b^ Shown are the detection results of the bacteria-spiked strawberry tissues in each media. The data are presented as the LS-means of the C_t_ (qPCR) and T_t_ (LAMP) values of each media at 21 h after incubating 10^4^ CFU/mL of *X*. *fragariae* with healthy strawberry tissues. The standard errors are 0.473 and 3.17 for qPCR and LAMP, respectively.

^c^ The effect of incubation of strawberry tissues with *X*. *fragariae* on detection is represented by the differences of the corresponding C_t_ (qPCR) or T_t_ (LAMP) values between (a) and (b).

#### (iii) Evaluation of the sample preparation procedure across X. fragariae strains and strawberry varieties

The effects of strawberry variety (note: this is a different set of varieties than used in the experiment describe in (i) above) and bacterial strain had no significant effect on detection at the 3 h or 21 h measurement periods according to qPCR and LAMP results (*p* > 0.05). However, paired *t*-tests showed significantly lower C_t_ and T_t_ values for 21 h versus 3 h measurements (*p* <0.0001). The mean difference was -1.05 and -1.87, for C_t_ and T_t_ values, respectively.

#### (iv) Sample preparation procedure vs. standard detection technique

Bacterial concentrations were statistically equivalent for inoculated strawberry samples processed through the new sample preparation procedure or the QIAGEN kit based on quantification from LAMP and qPCR, and results were not affected by sampling time (dai), tissue, or inoculum concentration (*p* > 0.05).

### Amplification specificity and detection limits of LAMP and qPCR primers

Both LAMP and qPCR primers did not amplify or only inconsistently amplified DNA of the non-target bacterial and fungal isolates listed in Table 2. When amplification occurred, T_t_ and C_t_ values were greater than T_t_ = 39 and C_t_ = 37 for LAMP and qPCR, respectively, which are typically higher than most threshold values commonly selected for these two procedures.

**Fig 2:**
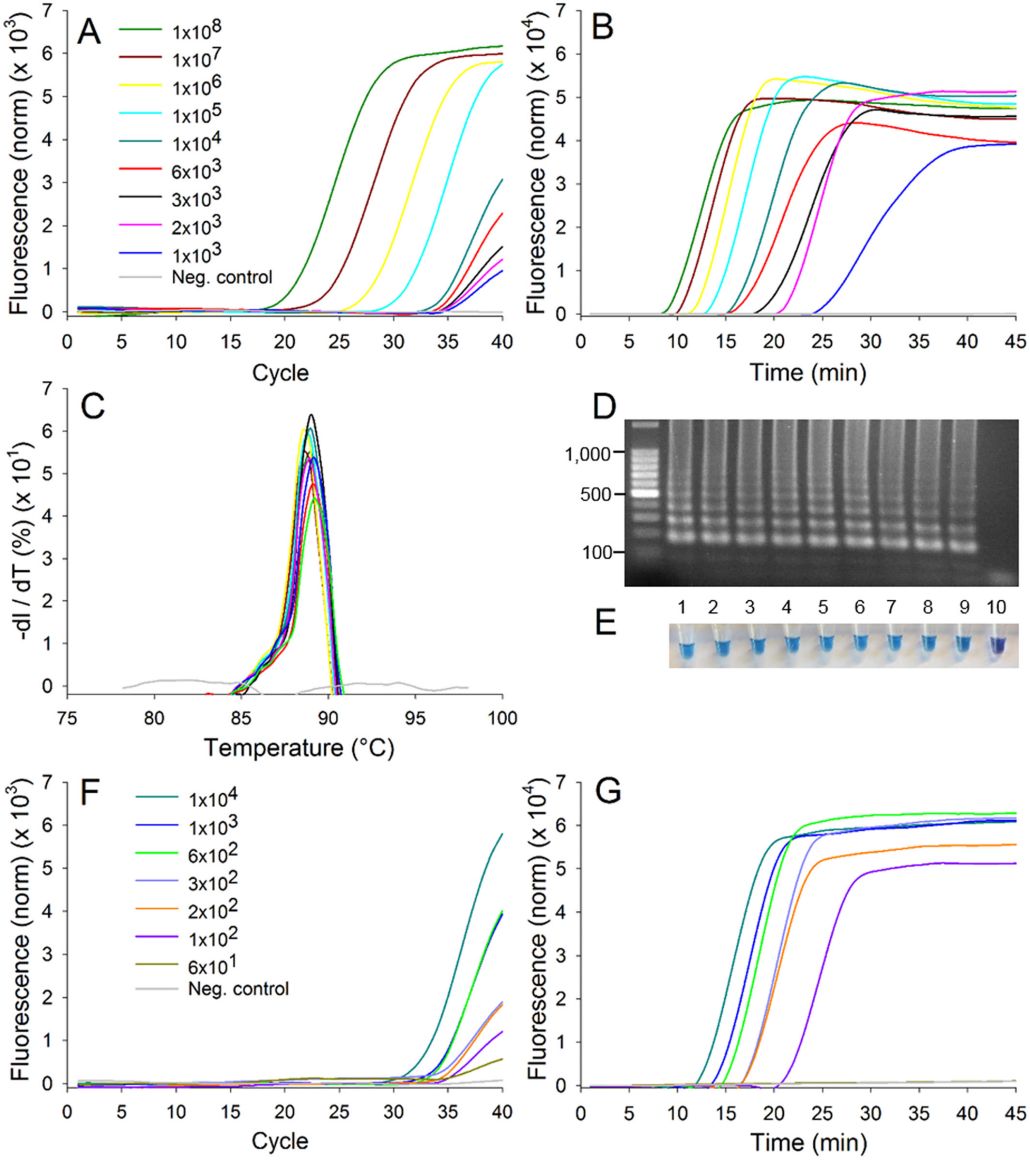
Reaction curves for qPCR and real-time LAMP. (A) qPCR and (B) LAMP amplification curves of pure bacterial DNA. (C) DNA melting curves for the LAMP assay in (B). (D) Gel electrophoresis results of the same samples processed in (B). Samples were run in 1.5% agarose gel with 100 bp DNA ladder and stained with ethidium bromide. (E) End product-detection with HNB dye of the same samples processed in (D). Samples 1–10 in (D) and (E) had the same concentration of *X*. *fragariae* as shown in the legend of (A) from top to bottom. (F) qPCR and (G) LAMP amplification curve of *X*. *fragariae-*spiked leaf samples processed through the sample preparation procedure.

The LAMP and qPCR primers did, however, amplify DNA from all *X*. *fragariae* isolates tested within 8 min and 22 cycles for LAMP and qPCR, respectively. For real-time LAMP reactions, SYTO^®^9 gave better amplification efficiency than SYBR^®^ Green I although it was not quantified. A single peak was observed in the LAMP melting curves of all the *X*. *fragariae* isolates, indicating specific amplification yielding the same DNA concatemers (Fig 2C). The typical ladder patterns of LAMP concatemers were shown in the agarose gel and the same DNA patterns were observed in all the positive samples only (Fig 2D). LAMP reactions with HNB dye yielded the same end results with T_t_ = 35 min as the real-time assays with T_t_ = 30 min (Fig 2E).

**Fig 3:**
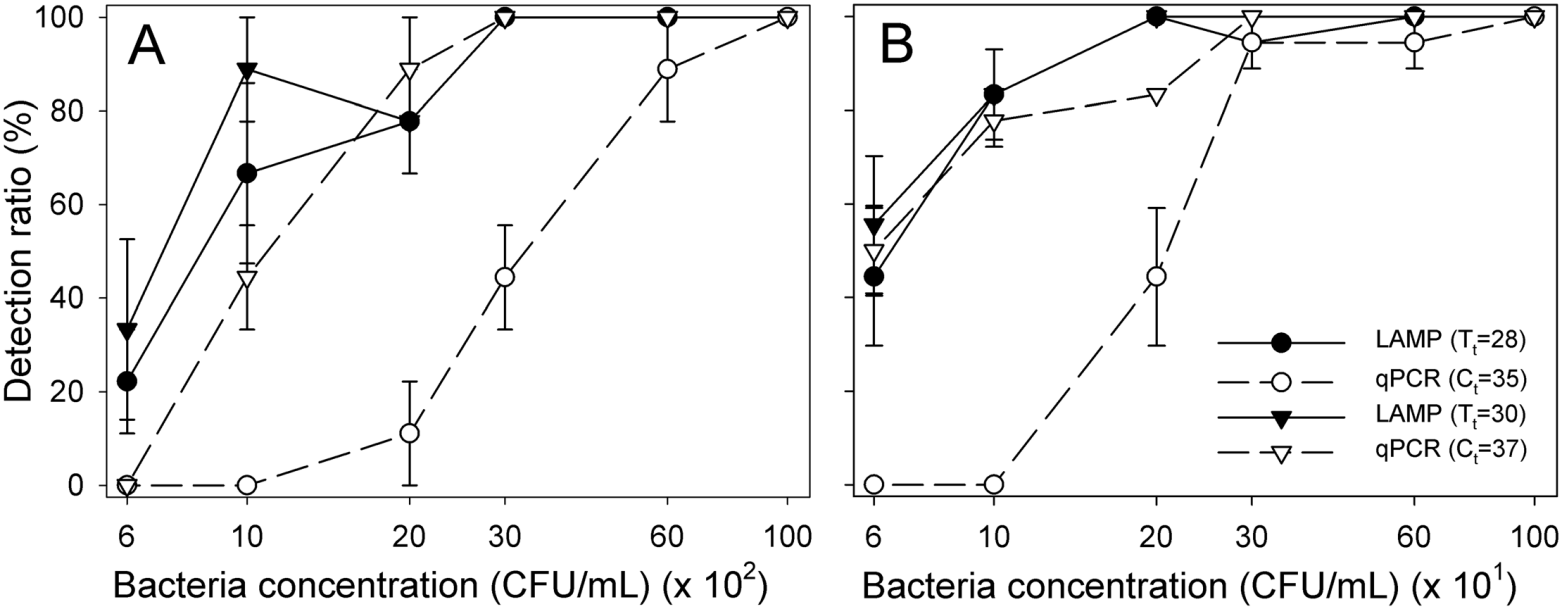
Detection rates of LAMP and qPCR. (A) DNA extracted from pure *Xanthomonas fragariae* cells. (B) DNA extracted from mixtures of *X*. *fragariae* cells and strawberry tissues after 21 h of incubation. Mean detection rates were calculated based on the percentage of positive samples at selected thresholds in 9 (3 samples per experiment) and 18 samples (6 samples per experiment) per bacterial concentration in (A) and (B), respectively. Error bars are standard errors of the means.

The detection limit of LAMP and qPCR was tested with pure *X*. *fragariae* DNA over a range of bacterial concentrations from 10^8^ to 10^3^ CFU/mL (Fig 2A and 2B). At the selected thresholds for LAMP (T_t_ = 28 min) and qPCR (C_t_ = 35 cycles), LAMP had lower detection limit (2×10^3^ CFU/mL, detected in 77.8% of the samples) than qPCR (6×10^3^ CFU/mL, detected in 88.9% of the samples) (Fig 3A). Increasing the thresholds to T_t_ = 30 min for LAMP and C_t_ = 37 for qPCR, the detection limit of LAMP was unchanged while that of qPCR improved to a value equivalent to LAMP (2×10^3^ CFU/mL, detected in 88.9% of the samples) (Fig 3A).

The detection limit for the samples processed through the sample preparation procedure was determined by adding live *X*. *fragariae* cells to healthy strawberry leaf or petiole tissue in PBS for the 21 h incubation period (Fig 2F and 2G). Bacteria-spiked leaf and petiole samples gave very similar results. Lower detection limits were observed with LAMP compared to qPCR with the previously mentioned thresholds. The starting concentration of *X*. *fragariae* before incubation ranged from 60 to 1000 CFU/mL. At conservative thresholds (T_t_ = 28 min, C_t_ = 35 cycle), LAMP and qPCR detected 300 and 600 CFU/mL of starting *X*. *fragariae* in 94.4% of the samples, respectively (Fig 3B). The detection limits of LAMP and qPCR were 100 and 200 CFU/mL in 83.3% of the samples, respectively, when the thresholds were T_t_ = 30 min and C_t_ = 37 cycles (Fig 3B). Samples with concentrations starting higher than 600 CFU/ml were detected 100% of the time for both assays. None of the negative control samples amplified in LAMP or qPCR.

### Sensitivity and specificity of the LAMP assay from field samples

**Fig 4:**
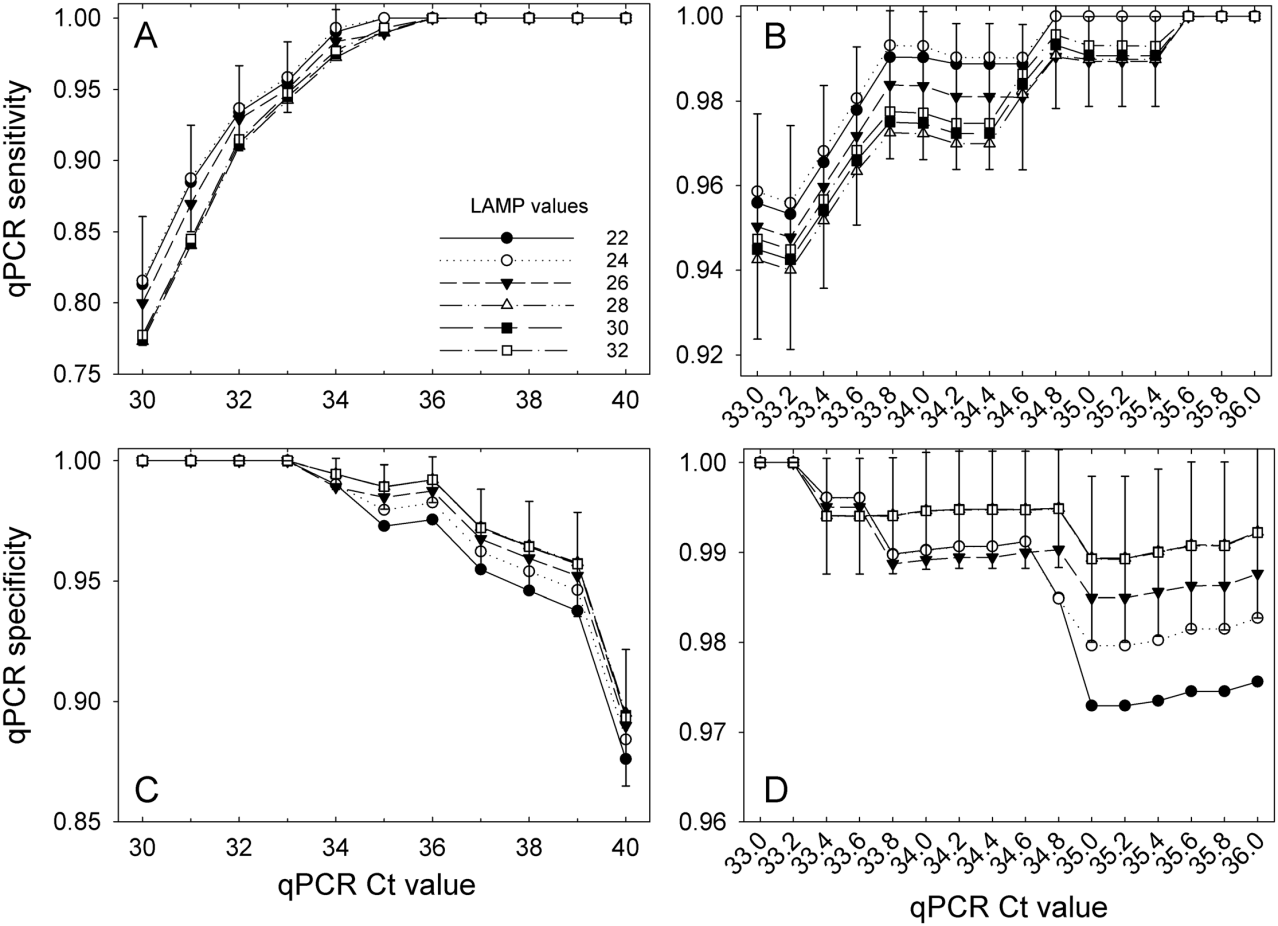
qPCR sensitivities (A, B) and specificities (C, D) relative to LAMP assay results. The sensitivities and specificities were estimated through latent class analyses for each combination of qPCR C_t_ and LAMP T_t_ threshold values. Error bars are standard errors of the means and are shown for a select series in each plot to avoid clutter. The series with the largest error was typically chosen as the representative series. Data points lacking error bars for that series were either too small or were not estimable by the MLE procedure.

**Fig 5:**
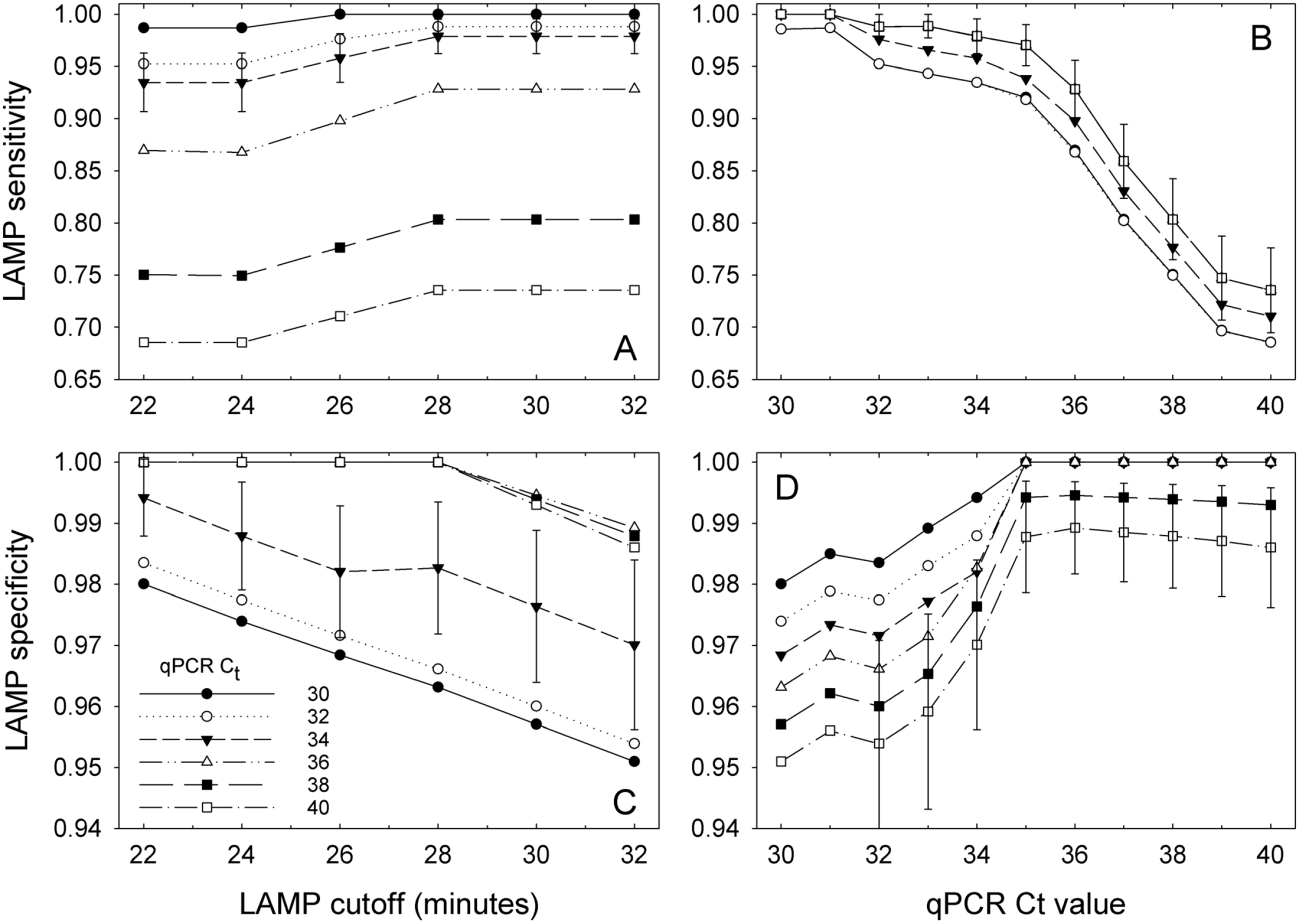
LAMP sensitivity (A, B) and specificity (C, D) relative to qPCR assay results. The sensitivities and specificities were estimated through latent class analyses for each combination of LAMP T_t_ and qPCR C_t_ threshold values. Error bars are standard errors of the means and are shown for a select series in each plot to avoid clutter. The series with the largest error was typically chosen as the representative series. Data points lacking error bars for that series were either too small or were not estimable by the MLE procedure.

**Fig 6:**
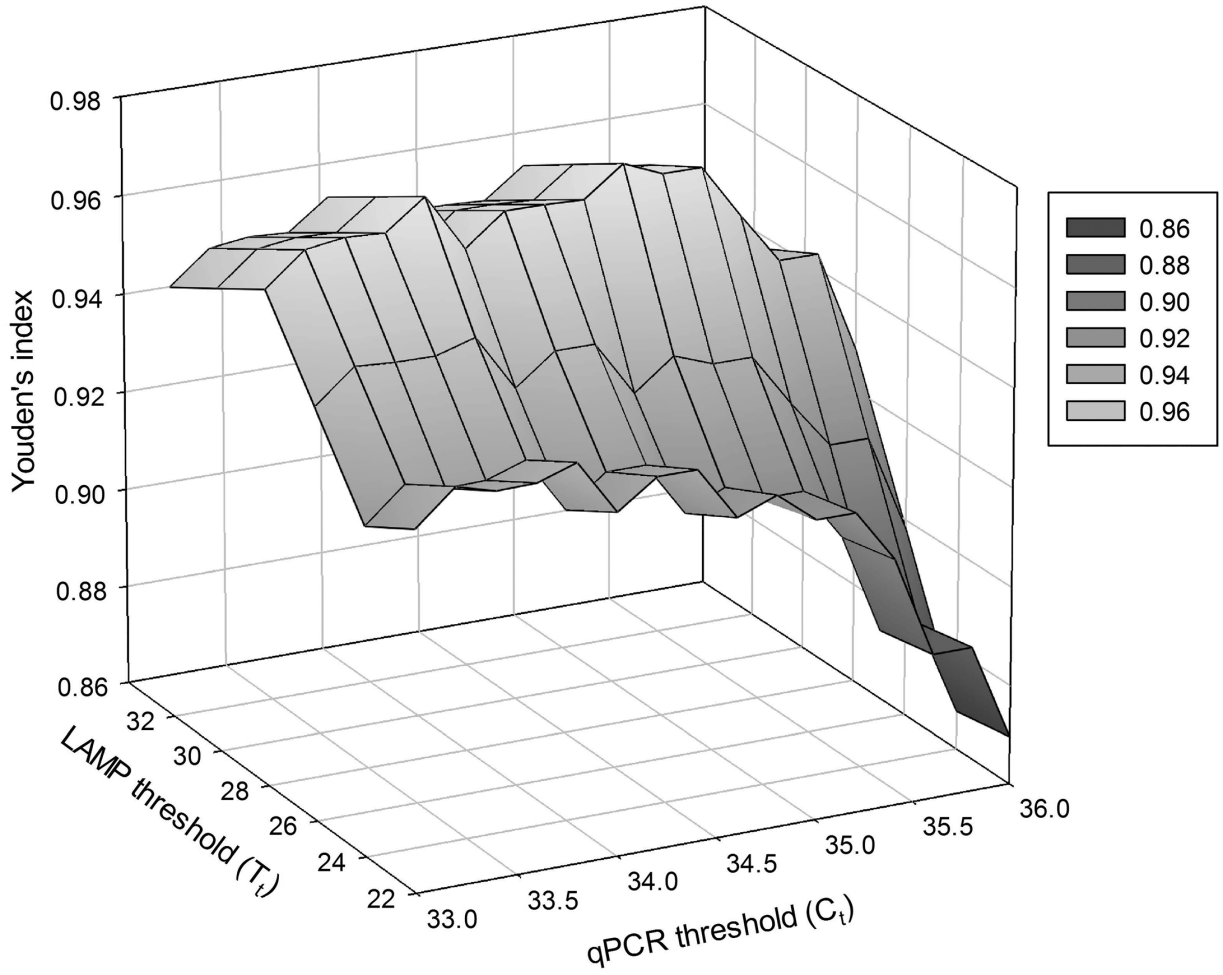
Youden’s index for the LAMP assay. The sensitivities and specificities used to calculate the index were estimated through latent class analyses for each combination of LAMP reaction times and qPCR C_t_ threshold values.

Although qPCR is not a true gold standard test, it was the test to which the LAMP assay was compared. The sensitivity of qPCR was equal to one at C_t_ values ≥36 and its specificity was equal to one at C_t_ values ≤33 irrespective of the LAMP threshold (Fig 4A and 4C). For C_t_ values between 33 and 36, qPCR sensitivity decreased and the specificity increased as the LAMP threshold increased (Fig 4B and 4D). Like qPCR, the sensitivity of the LAMP assay increased and its specificity decreased as the LAMP threshold increased from 22 to 32, but the rate of increase/decrease was much lower for LAMP than for qPCR (Fig 5A and 5C). It is interesting to note that for any given LAMP threshold, its sensitivity decreased and the specificity increased in response to an increasing qPCR C_t_ values (Fig 5B and 5D). This occurs because the proportions of the population that are categorized as being infected or not with *X*. *fragariae* are defined by the cross classified test results and not defined *a priori* by a reference or gold standard test. Thus, manipulating the threshold of one test while holding the other constant results in the characteristics changing for both tests. For sensitivity, if one test doesn’t capture the true positives well, like when the threshold is too low, the other test will have a near perfect sensitivity with its higher thresholds. Similarly, if one test doesn’t capture the specificity well, like when the threshold is too high, the other test will have near perfect specificity at its lower thresholds. Youden’s index was highest for LAMP values at 28 min and greater with the greatest value of 0.9737 observed at 28 min and C_t_ = 35 (Fig 6).

## Discussion

Angular leaf spot can cause significant losses in U.S. strawberry nursery production because of its quarantine status in European markets. Symptoms of *X*. *fragariae* are typically not encountered until the third or fourth year in the propagation cycle, although they are occasionally found in second year foundational plantings, which are typically established in greenhouses. Since nursery production of strawberry occurs in isolation from any large-scale commercial fruit production and the pathogen is known only to infect strawberry [2] and to not survive freely in soil [6], it is believed that the pathogen survives exclusively on existing nursery stock either as an endophyte or in a quiescent state. Recent experimental evidence indicates that the pathogen survives just underneath the epidermis in petiole tissue without causing symptoms [34]. Pathogenicity triggers may be associated with bacterial population density, environmental conditions, plant host characteristics or some combination of these or other unknown factors. Alternatively, it may be that the pathogen is producing very slight or uncharacteristic symptoms that simply escape detection. Whatever the case, it is important to utilize a sensitive detection protocol capable of detecting the pathogen early in production cycle so that infected stock can be rogued to minimize dispersal of the pathogen in the propagation chain.

Currently, the most sensitive and reliable methods for detecting *X*. *fragariae* are based on DNA detection methodologies that typically require a DNA extraction step followed by PCR. Most of the published protocols require specialized equipment, expensive reagents, and user-training [9–11] and, thus, can be prohibitively expensive for running large-scale screenings that might be necessary to survey a collection of nursery stock. In this study, we developed a LAMP assay to obtain a cost-effective and user-friendly method for *X*. *fragariae* detection *in planta*. Additionally, we developed a method of sample preparation to increase the likelihood of detection for situations when the bacteria density is expected to be low; it is not, however, a requirement of the LAMP assay that this procedure be used to prepare samples.

For the sample preparation procedure, we selected four media to test which of them would yield the best detection rate of *X*. *fragariae*. Over the 21 h incubation period, PBS was the most effective medium, showing greater sensitivity (i.e., lower C_t_ and T_t_ values) and enhanced detection of *X*. *fragariae* (Tables 3 and 4). It is likely that the enhancement seen with PBS was due to growth as a result of the bacteria utilizing nutrients released from strawberry tissues during incubation (Table 4). But measuring growth directly is difficult because the slow-growing *X*. *fragariae* are quickly over-grown by other bacteria present in the strawberry tissue suspension on agar plates, thus making colony counting impractical and likely inaccurate. Enhanced detection was also seen with SPB, one of the most commonly used nutrient-rich medium for *X*. *fragariae* [27]. However, the detection rate was lower for bacteria incubated in SPB than in PBS according to both LAMP and qPCR. The specific reason(s) for the reduction was not a focus of the study. However, possible explanations include the presence of PCR inhibitors (e.g., from tissue leachates, metabolites produced from leaf biota, or the general chemical composition of the media) or microbial competition. R2A also promoted large amounts of non-target bacteria growth, despite being a low-nutrient medium that has been used for isolation of *X*. *fragariae* from infected plants [29], which presumably competed with growth of *X*. *fragariae* and also yielded lower detection rates than PBS. The finding of PBS conferring greater target pathogen detection than nutrient-containing media has also been reported in animal studies [35, 36].

Distilled water, another medium without nutrients (like PBS), was the least effective of the four media tested. When the same concentration of *X*. *fragariae* was added to each of the media after incubation with healthy strawberry tissue, distilled water, like SPB, showed significantly higher C_t_ and T_t_ values than PBS and R2A (Table 4). This inhibitory effect could be attributed to the causes described above but, for water, it is also possible that the bacterial cells burst from osmotic pressure (hypotonic solution) over the 21 h incubation period in distilled water, which would lead to DNA release into the supernatant and subsequent loss during the centrifugation/concentration step of DNA extraction or by simple degradation.

With the sample preparation procedure, the detection limit of *X*. *fragariae* using LAMP was 200 CFU/mL, which is equivalent to 200 CFU per 100 mg of strawberry tissue for qPCR with C_t_ = 37. This limit is more sensitive than those reported previously for qPCR assays with similar C_t_ cut points for *X*. *fragariae* detection by Weller et al. [9], Turechek et al. [10], and Vandroemme et al. [11], which ranged from 300 to 1000 CFU per 100 mg of tissue. In these previous assays, bacterial DNA was extracted from strawberry tissue using commercial DNA extraction kits that allowed up to 100 mg of plant tissue per sample.

One of the motivations for developing the sample preparation procedure was to allow sampling of a greater proportion of tissue than what is common in many routine DNA extraction kits. The current sample preparation procedure was developed to accommodate sampling in the ratio of 1 g of strawberry tissue per 10 mL of PBS. Thus, the amount of tissue can be proportionally adjusted with the PBS volume to accommodate many different sampling scenarios. This ratio was determined to provide accurate results with minimal interference from PCR inhibitors. For spray-inoculated strawberry samples, LAMP and qPCR results were similar irrespective of whether the DNA was processed through the sample preparation procedure or through a commercial kit; in our case we mainly used the QIAGEN DNeasy Plant Mini Kit. This indicated that the sample preparation procedure performed as well as samples processed through a standard commercial kit for samples with uniformly distributed *X*. *fragariae*. Moreover, since the procedure allows sampling of a much larger amount of tissue (at least 10×), use of the procedure should increase the chance of detection for those real-life samples where bacteria are (spatially) heterogeneously distributed.

For LAMP reactions, the LAMP primers efficiently amplified all 40 *X*. *fragariae* isolates tested and exhibited excellent specificity by failing to amplify any of the other bacteria and fungi tested within the acceptable T_t_ threshold, including some closely-related *Xanthomonas* species and microbes associated with strawberry. Primers were not tested on *X*. *arboricola pv*. *fragariae* because it was not part of our available collection. Nevertheless, this pathogen is known only to occur in Europe and is currently not considered an important pathogen in U.S. strawberry production [37]. For real-time LAMP reactions, SYTO^®^9 dye yielded higher amplification efficiency than SYBR Green I, and this has been reported for different organisms in other studies [38]. The optimized recipe for the real-time LAMP assay developed in this study saved ~70% in cost compared to the commercial kit used in the LAMP assays for other Xanthomonas species [13, 16–18]. Real-time LAMP allows instant visualization of amplification results, is useful for the relative quantification of bacteria in samples, and helps to ensure specific amplification via melting curve analysis. Real-time LAMP can also be used with a portable fluorescence meter (e.g., Smart-DART^™^, Diagenetix Inc., Honolulu, HI) for field diagnosis; see reference [39] for an example.

On the other hand, LAMP with end point detection is simpler and less expensive to implement than real-time detection, making it more attractive to a wider range of users. In our assay, HNB dye was added prior to LAMP reactions to avoid cross-contamination among samples and eliminate the use of a UV illuminator. This contrasts to LAMP assays developed for other Xanthomonas species where fluorescent dyes were added to samples after the reaction [13, 17, 19]. LAMP with HNB dye provided end results equivalent to the real-time LAMP.

The sensitivity and specificity of the LAMP assay was measured through a latent class analysis. As mentioned above, LCA exploits the cross-classified results from two or more tests and uses a maximum likelihood approach to designate individual test results into two mutually exclusive categories (+ or −) and then uses this information to estimate each test’s characteristics. Inherent in this approach is the assumption that each of the two tests produce a binary outcome. This was not the case for either LAMP or qPCR. To overcome this problem, a factorial-like combination of cutoff points for both tests was used to evaluate their performances. For tests that produce a quantitative output, the selection of a cutoff can be somewhat arbitrary and is based on the type of error one is trying to minimize. For example, to avoid false negative decisions, selecting a threshold with a high sensitivity (e.g., a C_t_ value of 37 or a T_t_ value of 30) would accomplish the goal but at a cost of a greater false positive rate. Hence, the analysis conducted offers the reader the opportunity to analyze the outcome over a range of combinations. In addition, the selection of multiple thresholds was done to alleviate some of the issues associated with the dichotomization of a continuous variable [40].

Using the T_t_ and C_t_ thresholds identified as optimal through the latent class analysis and Youden’s index (28 and 35, respectively), LAMP had detection limits 2–3 times lower than qPCR for detecting both pure bacterial cells and from bacteria-spiked strawberry samples. When the T_t_ and C_t_ thresholds were increased to 30 and 37, respectively, qPCR’s detection limit improved and was equivalent to the LAMP assay, which was unchanged, for pure bacterial cells at 2×10^3^ CFU/mL. This is 2.5–5 fold lower than those reported in previous qPCR studies with C_t_ thresholds of ~37: 10^4^ CFU/mL in Turechek et al. [10] and 5×10^3^ CFU/mL in Vandroemme et al. [11]. With the higher thresholds, both LAMP and qPCR had lower detection limits for samples prepared from bacteria-spiked strawberry tissues, with LAMP being twice as sensitive as qPCR, and 3–10 fold more sensitive than those reported in previous qPCR assays [9–11].

In addition to being more sensitive than qPCR at select thresholds, LAMP is also easier to perform and can be done more quickly than qPCR. For example, amplification of DNA from 10^8^ CFU/mL of *X*. *fragariae* using LAMP with SYTO^®^9 dye could be detected within 8 min from the start of the reaction, compared to 25 min for qPCR. LAMP reactions are conducted at a constant temperature, thus it does not require stringent thermal cycling nor an expensive qPCR machine, making it more cost-effective and applicable. Moreover, LAMP reactions generate large amounts of pyrophosphate ion byproduct which interacts with magnesium ions in the reaction mixture to allow colorimetric end-point detection using a metal-ion detector, such as HNB dye, to further reduce the cost [25]. However, real-time LAMP was found to be less accurate in DNA quantification–with *R*^2^ of 0.910 for LAMP vs. 0.998 for qPCR for their linear standard curves–of which similar observation has been reported [13, 18]. If absolute quantification is not the major concern, then LAMP is a much more feasible tool than qPCR for low-cost detection and on-site diagnosis.

In conclusion, the LAMP assay with the sample incubation and crude DNA extraction procedure could serve as a sensitive and cost-effective method for detection of low quantities of *X*. *fragariae* in strawberry nursery stock. Detection at the early stages of production is essential in order to apply effective disease management strategies–usually plant removal–in efforts to reduce economic losses associated with quarantine violations.
